# Poultry Drinking Water Used for Avian Influenza Surveillance

**DOI:** 10.3201/eid1309.070517

**Published:** 2007-09

**Authors:** Y.H. Connie Leung, Li-Juan Zhang, Chun-Kin Chow, Chun-Lok Tsang, Chi-Fung Ng, Chun-Kuen Wong, Yi Guan, J.S. Malik Peiris

**Affiliations:** * The University of Hong Kong, Hong Kong Special Administrative Region, People’s Republic of China; 1 These authors contributed equally to this article.

**Keywords:** Avian influenza, surveillance, poultry, drinking water, dispatch

## Abstract

Samples of drinking water from poultry cages, which can be collected conveniently and noninvasively, provide higher rates of influenza (H9N2) virus isolation than do samples of fecal droppings. Studies to confirm the usefulness of poultry drinking water for detecting influenza (H5N1) should be conducted in disease-endemic areas.

Pandemic influenza originates from influenza viruses of birds (*1*). Live poultry markets play a crucial role in maintenance, amplification, and dissemination of avian influenza viruses (*2**–**4*) and are a risk factor for zoonotic transmission of highly pathogenic avian influenza (H5N1) viruses to humans (*5**,**6*). Maintaining surveillance of live poultry markets for influenza viruses is therefore important. In routine surveillance of live poultry markets, handling birds for collecting tracheal or cloacal swabs is often unacceptable to the bird sellers. Because avian influenza viruses were believed to be transmitted primarily by the oral–fecal route (*7*), fecal droppings were therefore regarded as the noninvasive specimen of choice for surveillance purposes (*8*). However, emerging evidence from experimental studies indicates that H9N2 (*9*) and H5N1 (*10*) subtypes are shed in higher titers in the upper respiratory tract. We tested the hypothesis that sampling drinking water is a convenient, noninvasive, and sensitive method for conducting avian influenza surveillance in live poultry markets. Because vaccine-derived Newcastle disease virus (NDV) is also commonly isolated from poultry in Hong Kong, we used NDV isolation rates for comparison.

As part of our ongoing surveillance in live poultry markets in Hong Kong, 51–67 poultry stalls in 8 poultry markets were sampled monthly from August 2004 through July 2005. Typically, several poultry of the same type share a cage, and all birds in the same cage share a drinking water trough, which is intermittently filled from the municipal water supply. We collected paired samples: drinking water from the water trough supplying a cage and a fresh fecal dropping from the tray under that same cage. Because the numbers of minor poultry (poultry other than chickens) sampled during this period were smaller, we included additional data (413 paired specimens collected from August 2005 through November 2006) obtained from cages holding silkie chickens, guinea fowls, pigeons, chukars, and pheasants.

One fresh fecal swab and 0.5 mL of the drinking water were collected from each cage. The fecal dropping represents a sample from 1 bird, in contrast to the drinking water trough, which was shared by all the birds in the cage. A total of 2,503 specimen pairs were collected. The fecal sample swab and water sample were separately put into 1 mL of virus transport medium containing M199 (9.5 g/L), penicillin G (2 × 10^6^ U/L), polymyxin B (10 × 10^6^ U/L), gentamicin (2,500 mg/L), nystatin (0.5 × 10^6^ U/L), ofloxacin HCl (100 mg/L), and sulfamethoxazole (1 g/L). A 200-μL aliquot of each sample was inoculated into a 9- to 11-day-old embryonic egg and incubated at 37^o^C for 3 days. Harvested allantoic fluid was tested for hemagglutination by using turkey erythrocytes. Hemagglutination-positive isolates were subtyped by using hemagglutination inhibition and neuraminidase inhibition tests with reference antiserum (*11*).

Of the 2,503 specimen pairs, influenza (H9N2) was isolated from 207 chickens (overall isolation rate 8.3%), 24 fecal samples alone (isolation rate 1.0%), 174 drinking water samples alone (7.0%), and 9 fecal and drinking water pairs (0.4%) (Table 1). The isolation rate for fecal samples was significantly lower than that for drinking water samples (p<0.001). The influenza (H9N2) isolation rates in drinking water and fecal droppings for silkie chickens were 5.8% and 0.6%, respectively (p = 0.005); for pigeons these rates were 3.8% and. 0%, respectively (p = 0.01). Isolation rates from pheasant gave a similar trend, although the results were not statistically significant (p = 0.11). The specimen numbers from guinea fowl and chukars were too small to be meaningfully analyzed.

**Table 1:** Avian influenza (H9N2) virus in chickens (August 2004–July 2005) and minor poultry (August 2004–November 2006)*

Species	No. pos/no. tested (% pos)			
	Overall isolation rate	Feces only	Drinking water only	Feces and drinking water
Chicken	207/2,503 (8.3)	24/2,503 (1.0)	174/2,503 (7.0)	9/2,503 (0.4)
Minor poultry				
Silkie chicken	10/171 (5.8)	0/171 (0)	9/171 (5.3)	1/171 (0.6)
Guinea fowl	1/13 (7.7)	1/13 (7.7)	0/13 (0)	0/13 (0)
Pigeon	6/158 (3.8)	0/158 (0)	6/158 (3.8)	0/158 (0)
Chukar	1/23 (4.3)	1/23 (4.3)	0/23 (0)	0/23 (0)
Pheasant	10/48 (20.8)	2/48 (4.2)	7/48 (14.6)	1/48 (2.1)

*Pos, positive for avian influenza (H9N2) virus.

In contrast, the isolation rate for NDV in chickens, silkie chickens, guinea fowl, and chukars did not show a comparable result (Table 2). In fact, for most of these species, the isolation rate from fecal samples was higher than that from drinking water samples, although these differences were not statistically significant.

**Table 2:** Newcastle disease virus in chickens (August 2004–July 2005) and minor poultry (August 2004–November 2006)*

Species	No. pos/no. tested (% pos)			
	Overall isolation rate	Feces only	Drinking water only	Feces and drinking water
Chicken	95/2,503 (3.8)	53/2,503 (2.1)	33/2,503 (1.3)	9/2,503 (0.4)
Minor poultry				
Silkie chicken	8/171 (4.7)	6/171 (3.5)	2/171 (1.2)	0/171 (0)
Guinea fowl	2/13 (15.4)	2/13 (15.4)	0/13 (0)	0/13 (0)
Pigeon	4/158 (2.5)	2/158 (1.3)	2/158 (1.3)	0/158 (0)
Chukar	1/23 (4.3)	1/23 (4.3)	0/23 (0)	0/23 (0)
Pheasant	0/48 (0)	0/48 (0)	0/48 (0)	0/48 (0)

*Pos, positive for Newcastle disease virus.

To estimate survival of influenza (H9N2) in water troughs, we inoculated subtype H9N2 into a trough containing drinking water taken from a poultry cage (i.e., a trough containing some organic debris rather than chlorinated water directly taken from the municipal supply). The virus titer soon after inoculation was 10^3.3^ 50% egg infectious doses/mL of water, which is comparable to the titer of virus in subtype H9N2–infected water troughs in the retail market setting (unpub. data). Virus could be isolated from the water trough at 8, 12, 24, and 48 h postinoculation but not at 56 or 72 h postinoculation. When the experiment was repeated with fresh tap water or distilled water, virus remained viable for 8 and 12 h, respectively. This finding suggests that virus may survive in drinking water troughs for 8–48 h, perhaps depending on level of chlorination and organic content of the water.

During our study, only subtype H9N2 viruses were isolated from Hong Kong’s poultry markets. The results from these field epidemiologic studies are compatible with data from experimental infection of poultry with subtype H9N2 viruses (*9*). Because titers of subtype H5N1 virus were higher in tracheal swabs than in cloacal swabs from ducks and other birds (*10**,**12*), subtype H5N1 virus isolation rates will likely be higher in drinking water than in fecal swabs, but this needs to be confirmed in studies conducted in regions where influenza (H5N1) is endemic. In contrast, NDV-infected chickens are reported to have virus detectable by reverse transcription–PCR for a longer period in the feces rather than the lungs (*13*), a finding consistent with our findings in live poultry markets.

The endemicity of highly pathogenic influenza (H5N1) in poultry in many countries across Asia and the continued detection of zoonotic transmission to humans, sometimes in regions where poultry outbreaks have not been reported, highlight the importance of systematic surveillance in live poultry markets. Systematic surveillance is especially important in regions where use of subtype H5 poultry vaccine is widespread. Whenever such studies have been conducted, previously unsuspected levels of virus activity have been found (*14*). Therefore, conducting such studies more widely, especially in areas known to be affected by subtype H5N1, is crucial. Such studies are the only way to determine the extent of virus transmission. They will also suggest potential interventions in the live poultry market systems that may effectively interrupt virus transmission in poultry; such interventions have been implemented in Hong Kong (*15*).

Our results provide evidence that taking samples from poultry drinking water troughs is an efficient way to conduct avian influenza surveillance. However, some caveats need to be noted. Drinking water potentially samples all the birds in a cage, whereas a fecal swab represents a single bird. Although the possibility for cage-to-cage transmission by infected water remains, NDV serves as a useful comparison in this regard. Different subtypes of avian influenza may have different shedding patterns from the respiratory tract compared with feces, and this strategy may not be applicable to all subtypes. Therefore, fecal droppings (or cloacal swabs) should also be collected. With these caveats accepted, sampling water from the drinking water troughs in poultry cages at live poultry markets and also at farms is likely to be a convenient, noninvasive, and practical strategy for implementing avian influenza surveillance for subtype H9N2 and perhaps also subtype H5N1; this approach should be evaluated in influenza-endemic regions.
